# Correction of malocclusion and oral rehabilitation in a case of amelogenesis imperfecta by insertion of dental implants followed by Le Fort I distraction osteogenesis of the edentulous atrophic maxilla

**DOI:** 10.1186/1472-6831-14-116

**Published:** 2014-09-17

**Authors:** Aysegul Apaydin, Bulent Sermet, Sevin Ureturk, Abdulsamet Kundakcioglu

**Affiliations:** Department of Oral and Maxillofacial Surgery, Faculty of Dentistry, University of Istanbul, Istanbul, Turkey; Department of Prostodontics, Faculty of Dentistry, University of Istanbul, Istanbul, Turkey; Department of Orthodontics, Faculty of Dentistry, University of Istanbul, Istanbul, Turkey; Istanbul Universitesi, Dıs Hekimligi Fakultesi, Cene cerrahisi, Capa, Sakayik sok., Buket apt, 39/2, No 27 Da 3, Nisantasi Istanbul, Turkey

**Keywords:** Amelogenesis imperfecta, Le Fort I Distraction osteogenesis, Edentulous maxilla, Dental implants, Malocclusion

## Abstract

**Background:**

Amelogenesis imperfecta refers a group of hereditary diseases affecting the teeth and can present a variety of clinical forms and appearances, compromising esthetic appearance. Amelogenesis imperfecta variably reduces oral health quality and can result in severe psychological problems.

**Case presentation:**

We present the management of an amelogenesis imperfecta Angle class III malocclusion case with speech, esthetics and functional problems. This is an example of the rarely presented delayed eruption with multiple morphologic dental alterations and edentulous maxilla.

There are only a few available reports in which this method is used method to correct sagittal discrepancies in edentulous patients.

Our treatment plan consisted of a preoperative diagnostic and prosthodontics phase (including preparation of guiding prosthesis), followed by a surgical phase of Le Fort I osteotomy, distraction osteogenesis to correct the malocclusion, implant insertion and a follow up final restorative phase.

**Conclusions:**

Our treatment strategy attempts to serve patient needs, achieving function and esthetics while also minimizing the risk of reconstruction failure. Treatment not only restored function and esthetics, but also showed a positive psychological impact and thereby improved perceived quality of life.

## Background

Amelogenesis imperfecta (AI) refers to a group of hereditary disorders characterized by defective formation or calcification of enamel, normally resulting in primary and permanent dentitions. Based on clinical and radiographic findings, as well as hereditary criteria, AI can be broadly classified as either hypo-plastic, hypo-calcified or hypo-maturation. Using a combination of clinical, radiographic and histological data, together with genetic criteria, Winter and Witkop define at least twelve distinct types of AI [1–3]. The average global prevalence of AI is 0.5% but epidemiologic studies have shown that the reported prevalence varies globally: % 0,43 (Turkey) [4]; %0,14 (Sweden) [5]; %0,1 (Argentina) [6]; %0,012 (Israel) [7].

There are numerous challenges associated with the management of AI patients, including poor esthetics, sensitivity of teeth, loss of tooth substance, higher risk for dental caries and decreased occlusal vertical dimension, with clinical severity varying across AI subtypes.

Several reports have described cases of malocclusion, characterized by an anterior open bite [8, 9]. Rowley et al. reported that 20% of AI cases have severe anterior open bite, while 44% have vertical dysgnathia [8]. However, the association of AI and delayed eruption has been little studied [10, 11].

The best clinical management strategy for AI is to follow a detailed treatment plan towards the desired clinical outcome. This report presents a case of AI with Class III malocclusion and highlights the rare presentation of delayed eruption, multiple morphologic dental alterations and edentulous maxilla, for which we used Le fort I distraction osteogenesis. The management goal was to achieve a better and more aesthetically pleasing antero-posterior and vertical position for the maxillary and mandibular bones, in adequate oral and facial proportions, and to construct biomechanically favorable prosthesis to provide efficient masticatory function.

The clinical strategy consisted of a preoperative diagnostic and prosthodontics phase (including preparation of guiding prosthesis), followed by a surgical phase of Le fort I osteotomy, distraction, implant insertion and final follow up restorative phase.

## Case presentation

A 17 year old female patient was referred to our clinic from the department of pedodontics, with dental anomalies, speech difficulty, and esthetic and functional problems (Figure 1). At 12 years-old the patient was diagnosed with AI. Past medical history revealed no related syndrome or pathology, neither in the patient nor family members. No alternative developmental or environmental causes of enamel defects (e.g., osteogenesis imperfecta, cerebral palsy, mental retardation) were detected. The patient suffered no complaints of ophthalmic, dermatologic or skeletal problems. The status of the hair and nails were normal and systemic examination was non-remarkable.

Deciduous teeth had been previously extracted and retainers applied. Panoramic radiography records revealed numerous unerupted teeth and clinical investigation found that hypo calcified, ill-shaped teeth had been extracted at an earlier stage (Figures 2 and 3).

**Figure 1:**
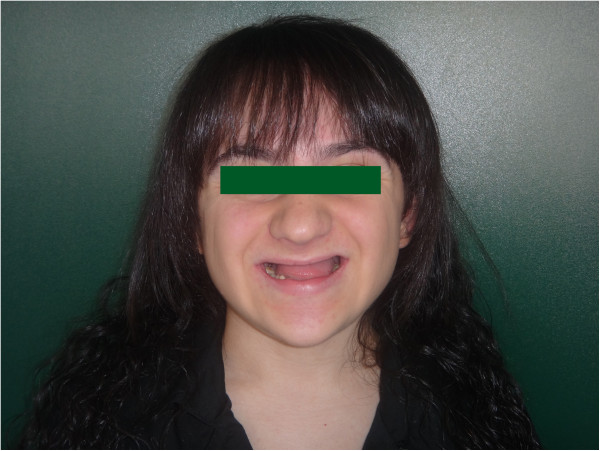
**Frontal view.**

**Figure 2:**
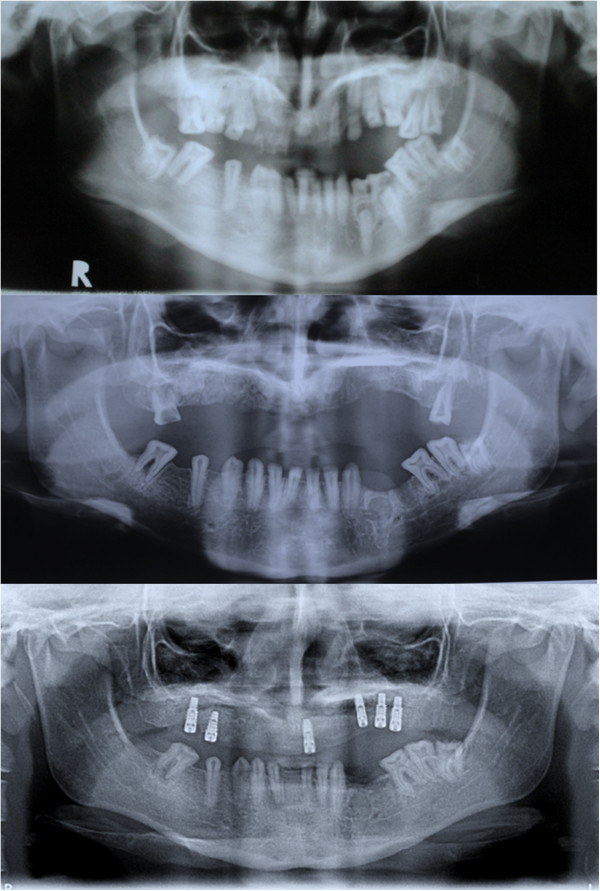
**Initial radiography (top), post extraction (middle) and implants in situ.**

**Figure 3:**
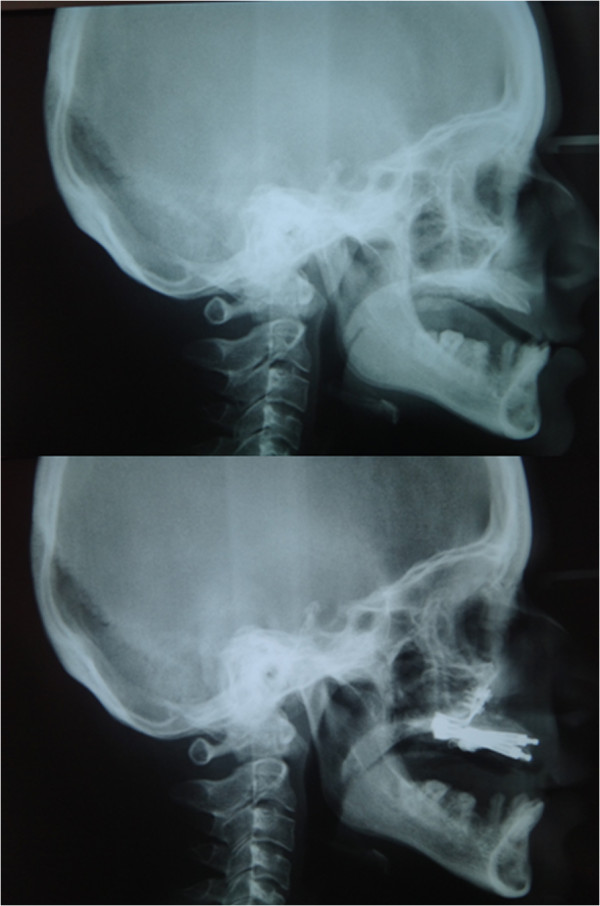
**Initial cephalography and distractors in situ.**

The patient and her family refused to have any extra tests of teeth and genetic counseling to confirm the diagnosis once more. She was sent to the pedodontics with that diagnosis for treatment and then to our clinics with the need of surgery.

Extra oral examination revealed a concave profile with pronounced chin and nose, short lower face and retrognathic maxilla (Figures 3 and 4). The temporomandibular joint, masticatory muscles and mouth opening were normal. Deviation, deflection, pain and discomfort were not detected during opening and closing, and there was no display of upper gingival tissue when smiling.

Intraoral examination identified an Angle Class III malocclusion with narrow, small and retrognathic maxilla and posterior bilateral cross bite. In the mandible 31, 32, 36, 37, 42–45 and 47 were present. Tooth crowns exhibited a distinct microform with large spacing, while the roots were normal in length and form (Figure 4). In maxilla, a large amount of alveolar bone loss was observed due to previous surgical interventions and gingival tissue was enlarged in the molar region. Posterior dentoalveolar height exceeded that of the anterior dentoalveolar. SNB values were high due to a decrease in the vertical dimension of the lower face. Skeletal growth was low angle with an S-N/Go-Me angle of 270 (ratio 71%). The narrow maxilla resulted in circular cross bite in the transversal dimension. There was no asymmetry in the face, with equal ramus and corpus dimensions on each side. The sagital dimension was of skeletal class III (ANB, 40; Wits-appraisal, 9 mm) with molars showing class III malocclusion. The maxilla was small and atrophic and cranial base length was 63 mm (S-N). Soft tissue examination found that the upper lip showed more retrochelie than the lower lip (-4 and -1 respectively) and that the lower lip was thicker than the upper lip.

**Figure 4:**
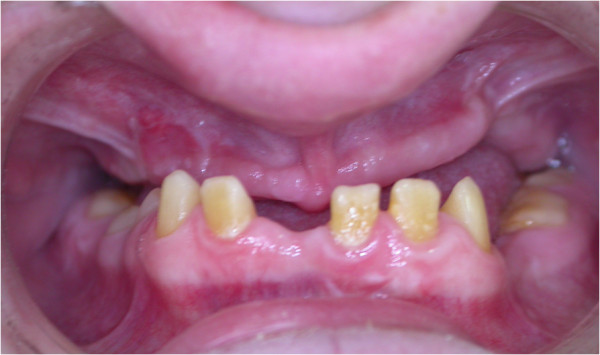
**The intraoral view.**

The principal treatment aims of improving function and facial/dental esthetics could be divided into skeletal (correct the class III pattern and vertical relationship) and dental (correct anterior open bite, achieve ideal overbite and over jet using dental implants) goals. Le Fort I advancement was selected for maxilla treatment, using distraction osteogenesis to achieve a stable result and prevent relapse.

Following Le fort I osteotomy, intraoral distractors (that had been previously adapted to the patient’s scull model) were placed at both sides of the maxilla (Figures 5 and 6). The maxilla was distracted for 10 days (0.5 mm × 2 per day) and, after a consolidation period, distractors were removed and implants inserted to the final sites. Implant sites with sufficient bone material had been previously selected using computed tomography (CT) data. The post-operative period was uneventful, with only slight paraesthesia and difficulty in mouth opening, which disappeared within two weeks. Patient’s initial and postoperative cephalograms are superimposed and 6 mm advancement of A point, 8 mm advancement of ANS (anterior nasal spina) point, 5 mm advancement of the upper lip, and 2 mm advancement of the tip of the nose were achieved (Figure 7). Finally, prosthetic restorations were applied to achieve an esthetic and functional result (Figure 8).

**Figure 5:**
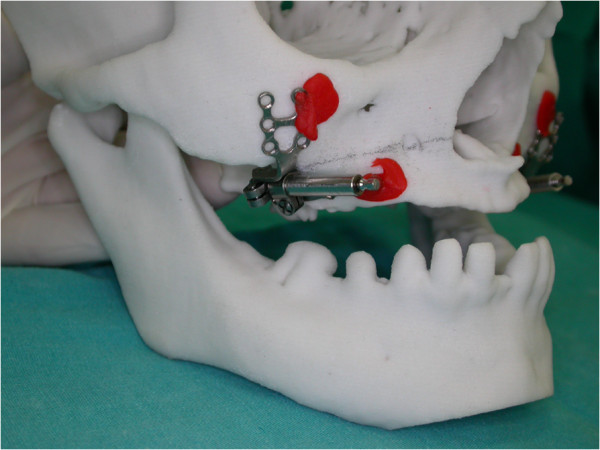
**Adapted distractors on the skull model.**

**Figure 6:**
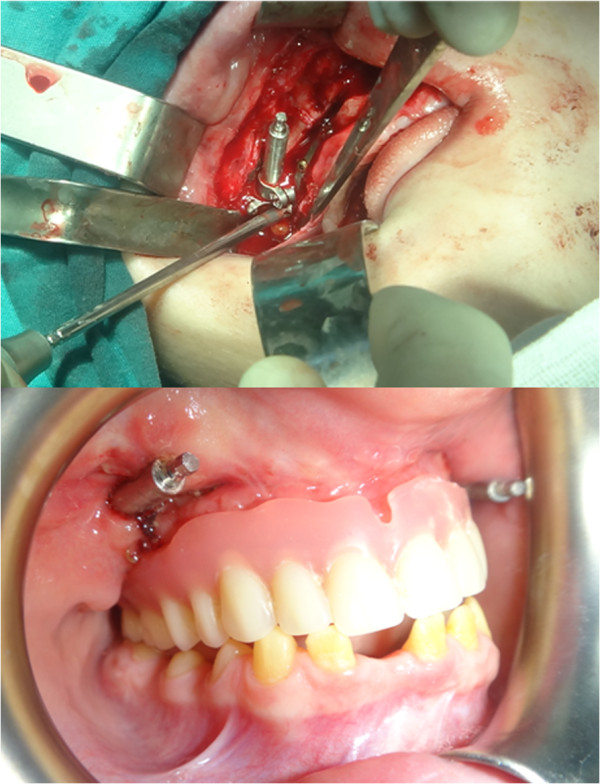
**Placement of distractors (top) and distraction period with prosthetic guidance.**

**Figure 7:**
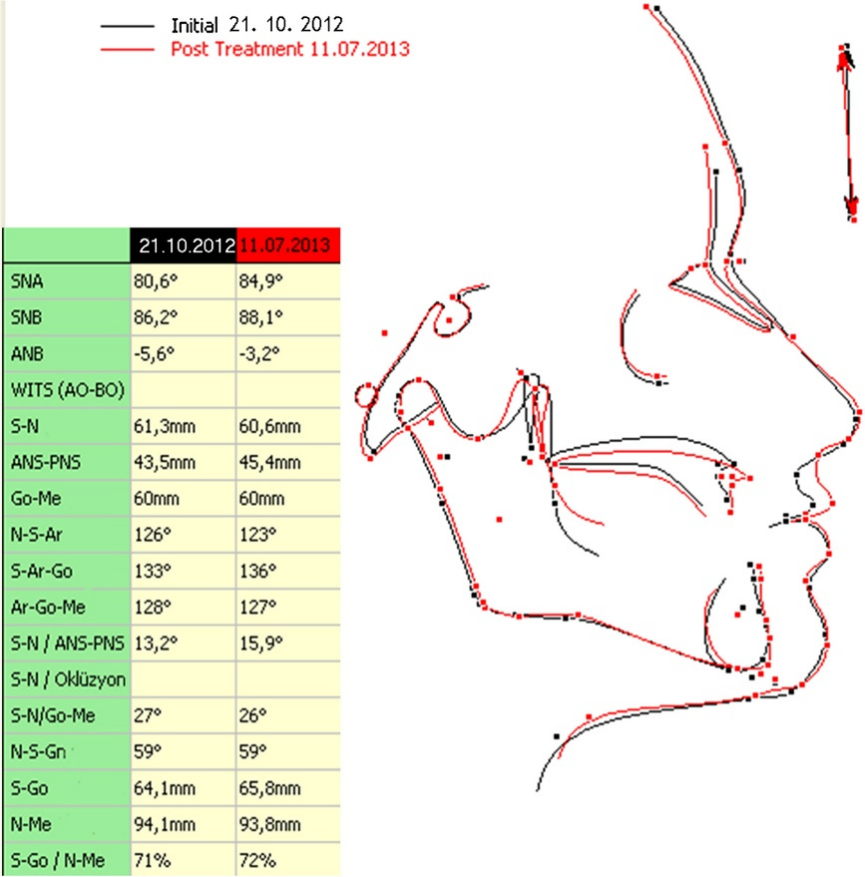
**Table and total superimposition.**

**Figure 8:**
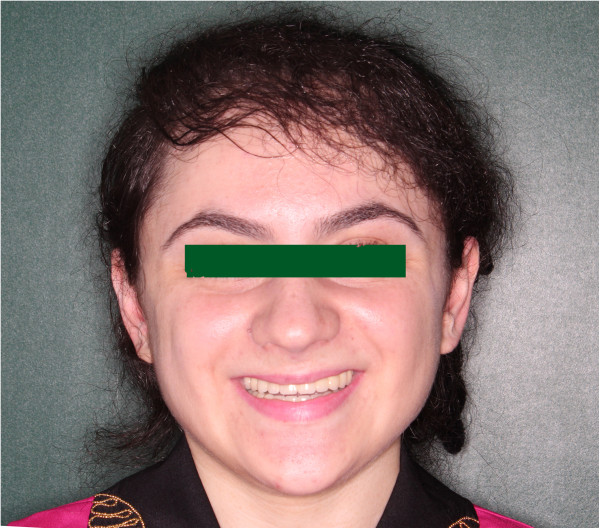
**Final outcome.**

## Discussion and conclusions

Amelogenesis imperfecta can have different inheritance patterns depending on the gene that is altered. Mutations in the ENAM gene are the most frequent known cause and are most commonly inherited in an autosomal dominant pattern. This type of inheritance means one copy of the altered gene in each cell is sufficient to cause the disorder.

Recent genetic studies suggest that the cause of a significant proportion of amelogenesis imperfecta cases remains to be discovered.

Treatment of AI is not only important for functionality but also for patient psychosocial health and esthetics. Although AI is a low prevalence condition, affected patients suffer a great number of clinical problems that affect their quality of life. In most cases the partial or total absence of enamel is associated with pain caused by thermal end chemical stimuli. Reduced crown due to incomplete eruption results in reductions to both masticatory function and occlusal vertical dimension. Considering all potential clinical complications and psychosocial affects, precise and early diagnosis of AI and proper treatment is vitally important for every patient.

Treatment may range from no intervention (mild cases) to full or partial mouth restoration (moderate and severe cases). In cases of severe penetrance of the hypomature or hypocalcified type/impacted teeth it may be most cost effective to edentulate the unrestorate dentition and rehabilitate with implant supported restorations from the outset of skeletal maturity [12]. The absence and shape of teeth and problems associated with enamel (such as sensitivity, staining and roughness) can be of major psychological and functional concern to AI patients. As such, the principal goals of the treatment plan should include pain managements, prevention, stabilization, restoration of any defects, and maintenance of aesthetics and function [13].

Modern management of AI requires a multidisciplinary approach utilizing a full armamentarium of disciplines [9, 14–16]. The management of teeth begins in the primary dentition, followed by the secondary dentition as the teeth erupt. Treatment protocols may change according to the clinical features of AI and are dependent on the absence/form of existent teeth. Management will also vary according to the needs of the patients and proposed treatment plans may consist of: 1) Surgical exposure followed by orthodontics extrusion of teeth and a restorative approach; 2) removable acrylic over denture; 3) cast overlay denture; or 4) implant insertion and fix prosthesis [15, 17].

The patient’s main concerns were difficulty in chewing and esthetics. Considering the patient’s relatively young age we aimed for a more stable outcome; Le fort I distraction osteogenesis would be more effective than classical Le Fort I advancement. In this case, the quality and volume of bone represented additional challenges to manage. Since patient didn’t accept any autogenic bone replacement and any further operations, it was exceedingly difficult to identify a proper and suitable location for a dental implant to support the prosthesis.

We have proposed that correction of the interalveolar relationship between the maxilla and the mandible can be achieved with distraction osteogenesis through orthognathic surgery and insertion of dental implants. Although insufficient bone volume allowed only a limited number of implants (and therefore prosthetic planning could not be extensive), the patient was satisfied with the final result, both esthetically and functionally.

The use of dental implants in edentulous patients provides safety and function in oral rehabilitation. For rehabilitation to be possible patients require adequate bone mass and suitable alveolar bone. These features can be achieved by augmenting bone graft (Onlay bone graft or inlay bone graft into the sinus). Another possibility, is to produce adequate bone mass and reposition alveolar bone by arranging the antero-posterior position of maxillary bone by Le Fort I down-fracture and inter-positional bone grafts. Some authors have used distraction osteogenesis in the severely resorbed maxilla, which can improve alveolar position and facilitate the jaw into a more favorable position [18, 19].

In most such those cases, Le Fort I distraction using an internal device is a stable and convenient option of correction. Indeed, distraction osteogenesis has been shown to be an accepted method of correcting sagittal discrepancies in cases of dentate and edentulous maxillary hypoplasia with stable long term results [20, 21]. However, there are only a few available reports in which this method is used to correct sagittal discrepancies in edentulous patients [21–23].

In atrophied maxilla, a lack of supporting bone can compromise the insertion of endoosseos implants. In cases of inadequate height and width of the maxillary alveolar crest a sufficient recipient site is required.

The benefits of distraction include avoidance of bone grafting and donor side morbidity, as well as its availability for use in surgery on younger patients and concurrent expansions of soft tissue envelops [24].

Treatment plans for AI are dependent on many factors including patient age, type and severity of disorder and intraoral conditions. Treatment should begin at childhood and continue into adolescence and consist of an interdisciplinary approach including periodontal, orthodontic, prosthodontics, surgical and restorative methods.

We report an orthognathic procedure for AI using Le Fort I distraction osteogenesis, in which a large difference was achieved between the initial and final profile of the upper lip, resulting in a greatly improved facial profile supported by prosthesis, with immediate improvement in chewing function and aesthetics.

It is often difficult to achieve stable and satisfactory results in the treatment of AI patients. This is exasperated in AI patients with rerouted maxilla. In such cases, pre-surgical orthodontics is often unachievable because of absence of teeth or lack of crown height and poor enamel condition.

Our treatment strategy attempts to serve patient needs, achieving function and esthetics while also minimizing the risk of reconstruction failure. When long term stability is in question, risks and benefits of the treatment plan should be thoroughly evaluated to achieve the best possible results according to the specific needs of each patient.

### Consent

Written informed consent was obtained from the patient for publication of this case report and any accompanying images. A copy of the written consent is available for review by the Editor-in-Chief of this journal.
